# Implementing the National Alzheimer’s Coordinating Center Uniform Data Set (v3) within the Diabetes Prevention Program Outcomes Study

**DOI:** 10.64898/2026.07.17.26357765

**Published:** 2026-07-21

**Authors:** Lindsay K Doherty, Isabella Dechiario, Hanna Sherif, Anna Bowers, Dianilka Martinez, Danurys L Sanchez, Gerardo J Febres, Owen Carmichael, Vallabh Shah, Neelesh K Nadkarni, Terry E Goldberg, James Noble, Jose A Luchsinger, Marinella Temprosa, DPPOS Research Group

## Abstract

**INTRODUCTION:**

The Diabetes Prevention Program (DPP) was a randomized clinical trial designed to prevent type 2 diabetes (T2D) in adults with prediabetes. The DPP Outcomes Study (DPPOS) is the 30-year follow-up of this cohort, focusing on T2D, prediabetes, and related complications. Cognitive assessments began in 2009 and expanded in 2022 to examine cognitive impairment, including Alzheimer’s disease (AD) and AD related dementias (ADRD), in the surviving cohort. To support these aims, the National Alzheimer’s Coordinating Center Uniform Data Set version 3 (NACC-UDSv3), the standardized framework used by Alzheimer’s Disease Research Centers, was implemented in DPPOS in 2022 to enable data sharing with NACC. These forms were complemented by cognitive tests administered in DPPOS. We aimed to integrate the NACC-UDSv3 into the existing longitudinal DPPOS framework while maintaining fidelity to its structure and developing automated reports to streamline cognitive outcomes adjudication.

**METHODS:**

Items from the 16 NACC-UDSv3 data forms were compared with those already collected within DPPOS to integrate overlapping similar items, add missing NACC-UDSv3 items, and create a dataset harmonized with NACC-UDSv3. Forms were adapted for electronic data capture (EDC) using the MIDAS (Multimodal Integrated Data Acquisition System, George Washington University). Automated reports integrated current and prior neuropsychological scores to support adjudications. In the first wave of the DPPOS-AD/ADRD study, 1561 cognitive adjudications were successfully completed using the harmonized DPPOS and NACC-UDSv3 data implemented into MIDAS.

**DISCUSSION:**

The DPPOS-AD/ADRD project demonstrated that NACC-UDSv3 can be successfully integrated into a long-standing longitudinal cohort not originally designed for AD/ADRD research. The harmonization, electronic capture, and automated adjudication processes may provide a practical framework for other cohorts seeking to incorporate NACC-UDSv3 to align with national AD/ADRD research standards.

## Full Text Availability

The license terms selected by the author(s) for this preprint version do not permit archiving in PMC. The full text is available from the preprint server.

